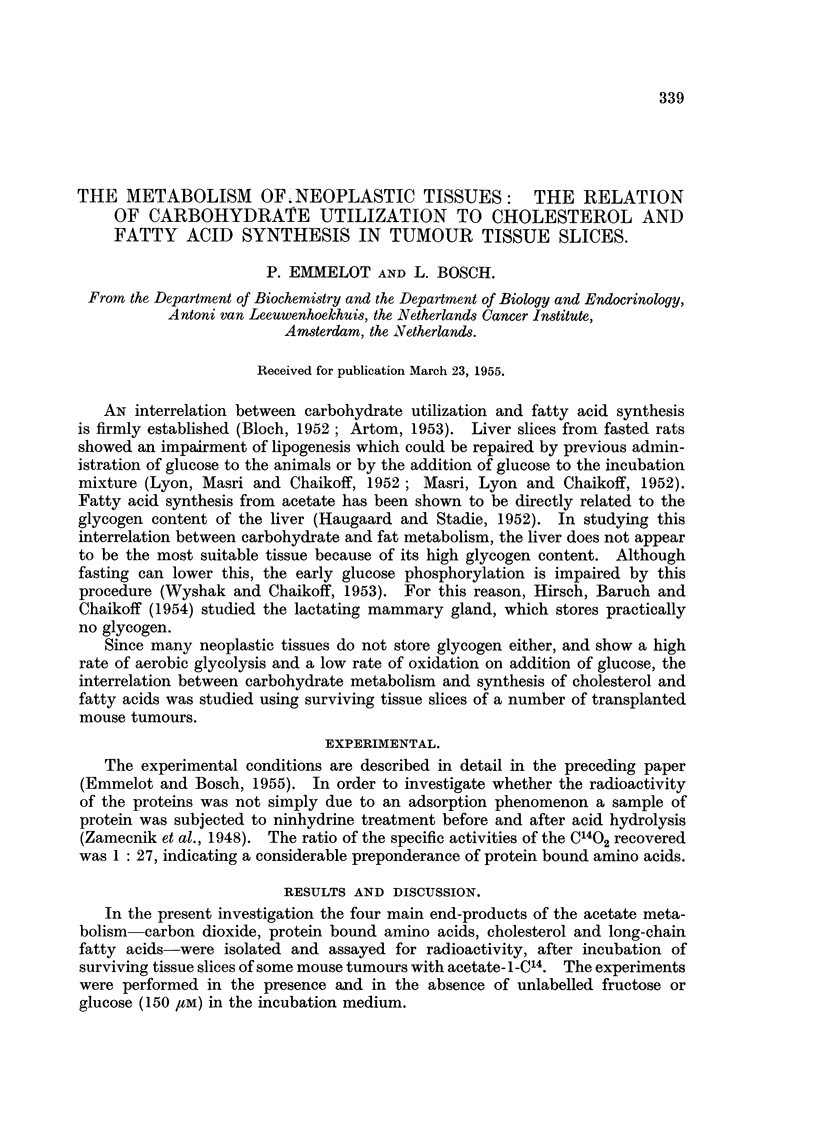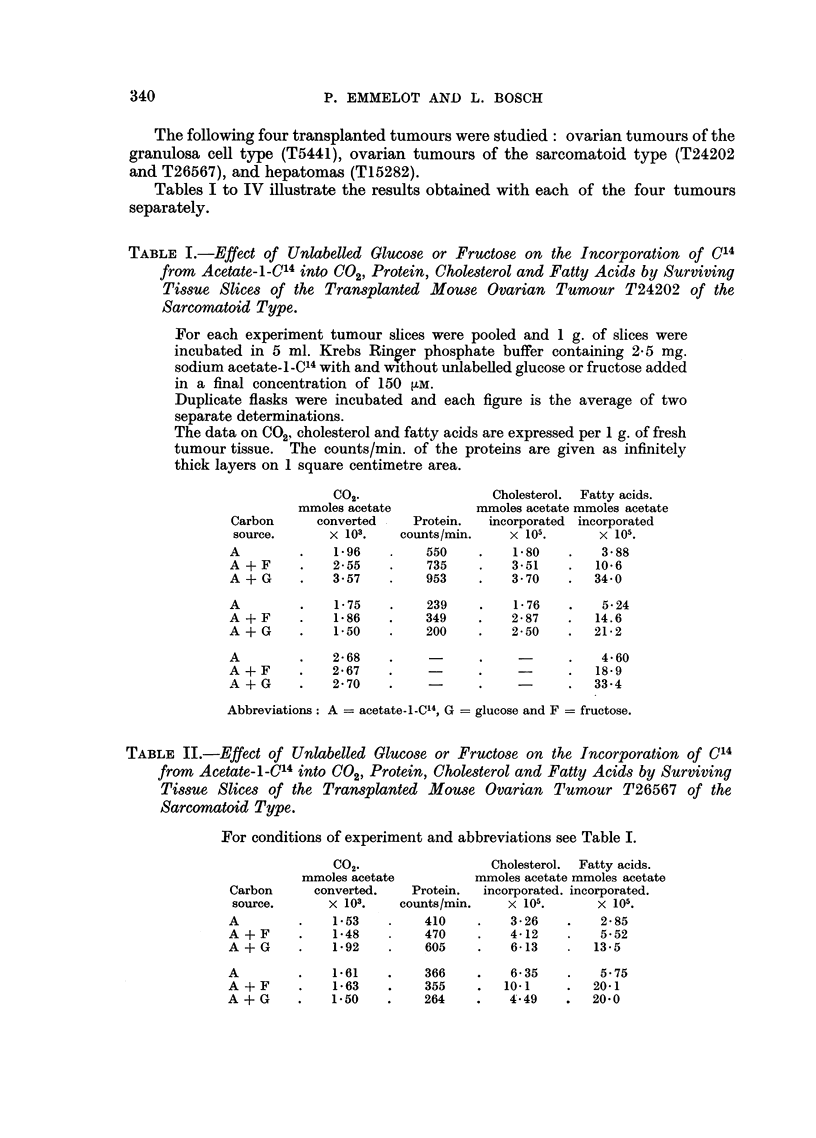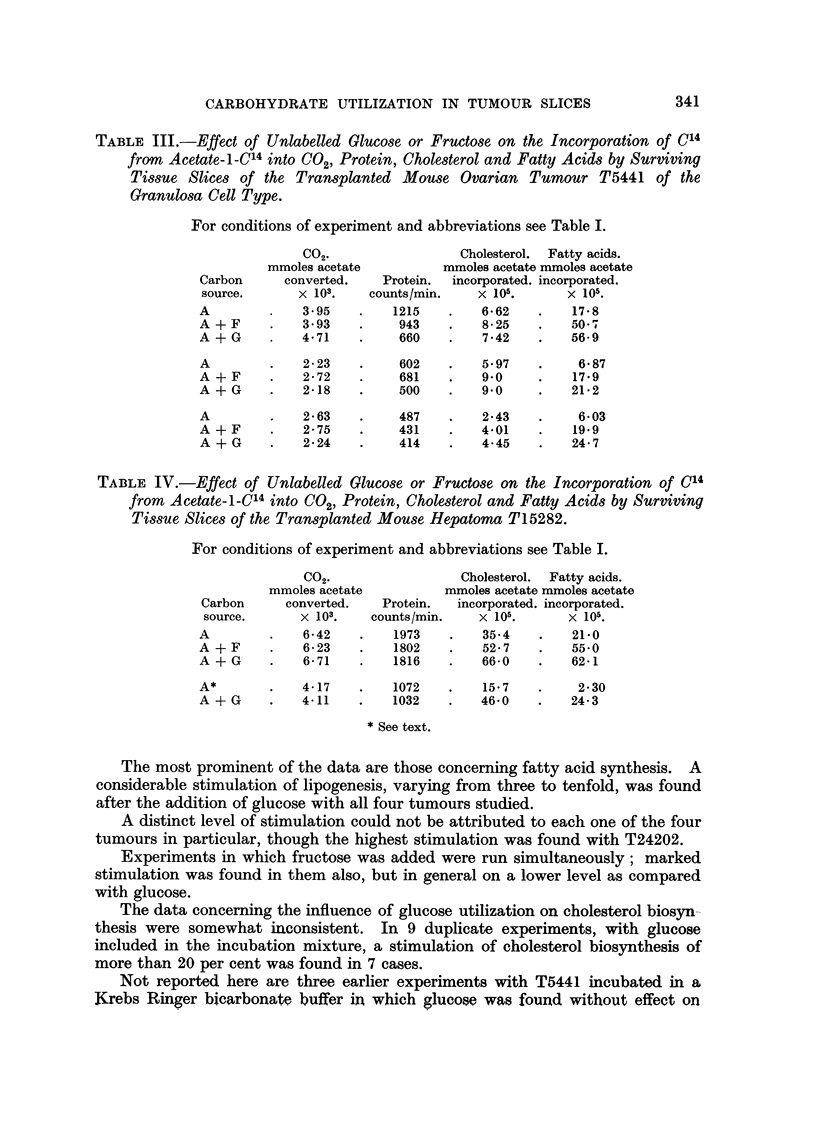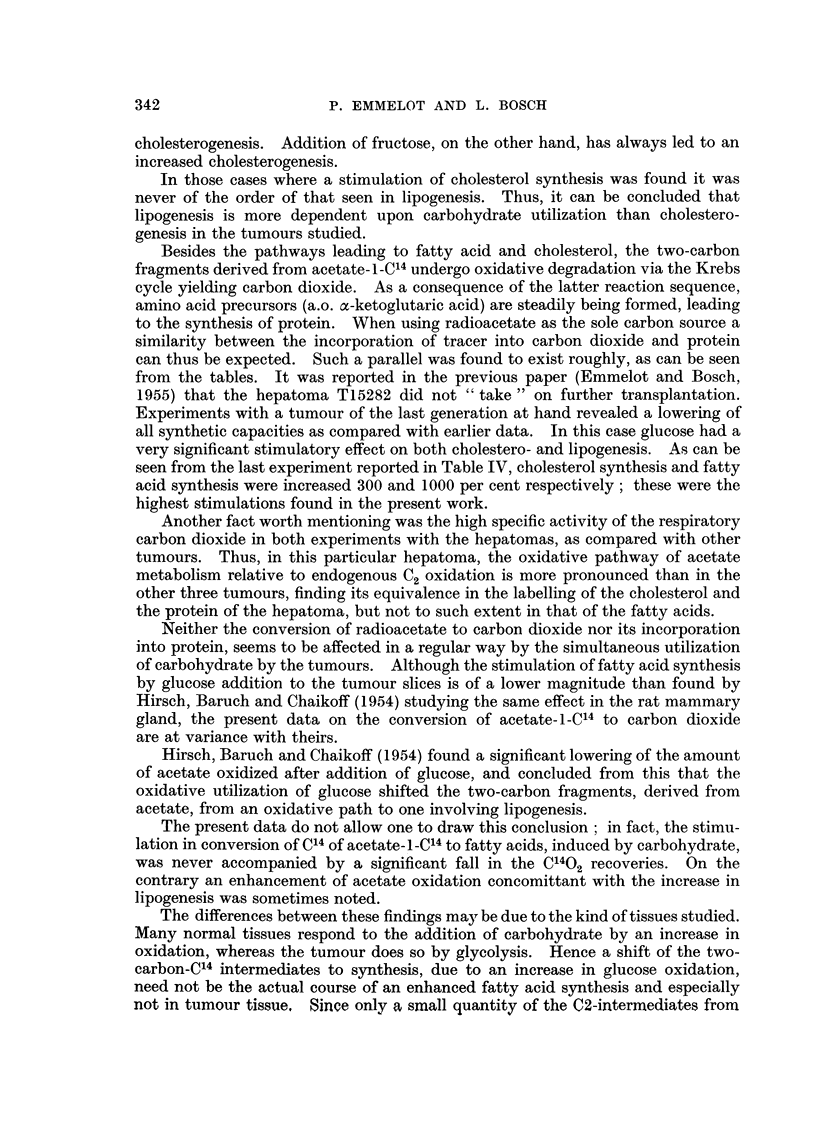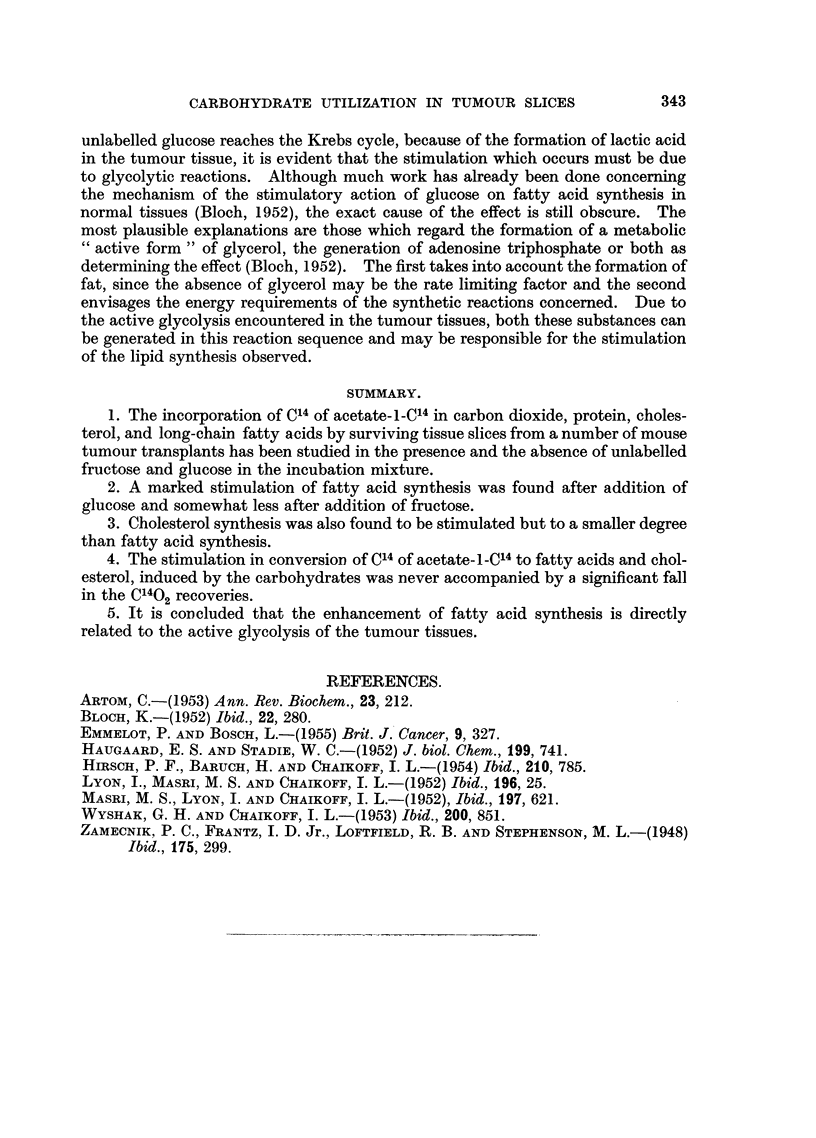# The Metabolism of Neoplastic Tissues: The Relation of Carbohydrate Utilization to Cholesterol and Fatty Acid Synthesis in Tumour Tissue Slices

**DOI:** 10.1038/bjc.1955.31

**Published:** 1955-06

**Authors:** P. Emmelot, L. Bosch


### THE METABOLISM OF NEOPLASTIC TISSUES: THE RELATION OF CARBOHYDRATE UTILIZATION TO CHOLESTEROL AND FATTY ACID SYNTHESIS IN TUMOUR TISSUE SLICES.

P. EMMELOT AND L. BOSCH.

*From the Department of Biochemistry and the Department of Biology and Endocrinology, Antoni van Leeuwenhoekhuis, the Netherlands Cancer Institute, Amsterdam, the Netherlands.*

Received for publication March 23, 1955.

AN interrelation between carbohydrate utilization and fatty acid synthesis is firmly established (Bloch, 1952; Artom, 1953). Liver slices from fasted rats showed an impairment of lipogenesis which could be repaired by previous administration of glucose to the animals or by the addition of glucose to the incubation mixture (Lyon, Masri and Chaikoff, 1952; Masri, Lyon and Chaikoff, 1952). Fatty acid synthesis from acetate has been shown to be directly related to the glycogen content of the liver (Haugaard and Stadie, 1952). In studying this interrelation between carbohydrate and fat metabolism, the liver does not appear to be the most suitable tissue because of its high glycogen content. Although fasting can lower this, the early glucose phosphorylation is impaired by this procedure (Wyshak and Chaikoff, 1953). For this reason, Hirsch, Baruch and Chaikoff (1954) studied the lactating mammary gland, which stores practically no glycogen.

Since many neoplastic tissues do not store glycogen either, and show a high rate of aerobic glycolysis and a low rate of oxidation on addition of glucose, the interrelation between carbohydrate metabolism and synthesis of cholesterol and fatty acids was studied using surviving tissue slices of a number of transplanted mouse tumours.

### EXPERIMENTAL.

The experimental conditions are described in detail in the preceding paper (Emmelot and Bosch, 1955). In order to investigate whether the radioactivity of the proteins was not simply due to an adsorption phenomenon a sample of protein was subjected to ninhydrine treatment before and after acid hydrolysis (Zamecnik *et al.*, 1948). The ratio of the specific activities of the $C^{14}O_2$ recovered was 1 : 27, indicating a considerable preponderance of protein bound amino acids.

### RESULTS AND DISCUSSION.

In the present investigation the four main end-products of the acetate metabolism—carbon dioxide, protein bound amino acids, cholesterol and long-chain fatty acids—were isolated and assayed for radioactivity, after incubation of surviving tissue slices of some mouse tumours with acetate-1-$C^{14}$. The experiments were performed in the presence and in the absence of unlabelled fructose or glucose (150 $\mu$M) in the incubation medium.

The following four transplanted tumours were studied : ovarian tumours of the granulosa cell type (T5441), ovarian tumours of the sarcomatoid type (T24202 and T26567), and hepatomas (T15282).

Tables I to IV illustrate the results obtained with each of the four tumours separately.

TABLE I.—*Effect of Unlabelled Glucose or Fructose on the Incorporation of $C^{14}$ from Acetate-1-$C^{14}$ into $CO_2$, Protein, Cholesterol and Fatty Acids by Surviving Tissue Slices of the Transplanted Mouse Ovarian Tumour T24202 of the Sarcomatoid Type.*

For each experiment tumour slices were pooled and 1 g. of slices were incubated in 5 ml. Krebs Ringer phosphate buffer containing 2·5 mg. sodium acetate-1-$C^{14}$ with and without unlabelled glucose or fructose added in a final concentration of 150 μM.

Duplicate flasks were incubated and each figure is the average of two separate determinations.

The data on $CO_2$, cholesterol and fatty acids are expressed per 1 g. of fresh tumour tissue. The counts/min. of the proteins are given as infinitely thick layers on 1 square centimetre area.

| Carbon source. | $CO_2$. mmoles acetate converted $\times 10^3$. | Protein. counts/min. | Cholesterol. mmoles acetate incorporated $\times 10^5$. | Fatty acids. mmoles acetate incorporated $\times 10^5$. |
|---|---|---|---|---|
| A | 1·96 | 550 | 1·80 | 3·88 |
| A + F | 2·55 | 735 | 3·51 | 10·6 |
| A + G | 3·57 | 953 | 3·70 | 34·0 |
| | | | | |
| A | 1·75 | 239 | 1·76 | 5·24 |
| A + F | 1·86 | 349 | 2·87 | 14.6 |
| A + G | 1·50 | 200 | 2·50 | 21·2 |
| | | | | |
| A | 2·68 | — | — | 4·60 |
| A + F | 2·67 | — | — | 18·9 |
| A + G | 2·70 | — | — | 33·4 |

Abbreviations : A = acetate-1-$C^{14}$, G = glucose and F = fructose.

TABLE II.—*Effect of Unlabelled Glucose or Fructose on the Incorporation of $C^{14}$ from Acetate-1-$C^{14}$ into $CO_2$, Protein, Cholesterol and Fatty Acids by Surviving Tissue Slices of the Transplanted Mouse Ovarian Tumour T26567 of the Sarcomatoid Type.*

For conditions of experiment and abbreviations see Table I.

| Carbon source. | $CO_2$. mmoles acetate converted. $\times 10^3$. | Protein. counts/min. | Cholesterol. mmoles acetate incorporated. $\times 10^5$. | Fatty acids. mmoles acetate incorporated. $\times 10^5$. |
|---|---|---|---|---|
| A | 1·53 | 410 | 3·26 | 2·85 |
| A + F | 1·48 | 470 | 4·12 | 5·52 |
| A + G | 1·92 | 605 | 6·13 | 13·5 |
| | | | | |
| A | 1·61 | 366 | 6·35 | 5·75 |
| A + F | 1·63 | 355 | 10·1 | 20·1 |
| A + G | 1·50 | 264 | 4·49 | 20·0 |

TABLE III.—*Effect of Unlabelled Glucose or Fructose on the Incorporation of $C^{14}$ from Acetate-1-$C^{14}$ into $CO_2$, Protein, Cholesterol and Fatty Acids by Surviving Tissue Slices of the Transplanted Mouse Ovarian Tumour T5441 of the Granulosa Cell Type.*

For conditions of experiment and abbreviations see Table I.

| Carbon source. | $CO_2$. mmoles acetate converted. $\times 10^3$. | Protein. counts/min. | Cholesterol. mmoles acetate incorporated. $\times 10^5$. | Fatty acids. mmoles acetate incorporated. $\times 10^5$. |
|---|---|---|---|---|
| A | 3·95 | 1215 | 6·62 | 17·8 |
| A + F | 3·93 | 943 | 8·25 | 50·7 |
| A + G | 4·71 | 660 | 7·42 | 56·9 |
| A | 2·23 | 602 | 5·97 | 6·87 |
| A + F | 2·72 | 681 | 9·0 | 17·9 |
| A + G | 2·18 | 500 | 9·0 | 21·2 |
| A | 2·63 | 487 | 2·43 | 6·03 |
| A + F | 2·75 | 431 | 4·01 | 19·9 |
| A + G | 2·24 | 414 | 4·45 | 24·7 |

TABLE IV.—*Effect of Unlabelled Glucose or Fructose on the Incorporation of $C^{14}$ from Acetate-1-$C^{14}$ into $CO_2$, Protein, Cholesterol and Fatty Acids by Surviving Tissue Slices of the Transplanted Mouse Hepatoma T15282.*

For conditions of experiment and abbreviations see Table I.

| Carbon source. | $CO_2$. mmoles acetate converted. $\times 10^3$. | Protein. counts/min. | Cholesterol. mmoles acetate incorporated. $\times 10^5$. | Fatty acids. mmoles acetate incorporated. $\times 10^5$. |
|---|---|---|---|---|
| A | 6·42 | 1973 | 35·4 | 21·0 |
| A + F | 6·23 | 1802 | 52·7 | 55·0 |
| A + G | 6·71 | 1816 | 66·0 | 62·1 |
| A* | 4·17 | 1072 | 15·7 | 2·30 |
| A + G | 4·11 | 1032 | 46·0 | 24·3 |

* See text.

The most prominent of the data are those concerning fatty acid synthesis. A considerable stimulation of lipogenesis, varying from three to tenfold, was found after the addition of glucose with all four tumours studied.

A distinct level of stimulation could not be attributed to each one of the four tumours in particular, though the highest stimulation was found with T24202.

Experiments in which fructose was added were run simultaneously ; marked stimulation was found in them also, but in general on a lower level as compared with glucose.

The data concerning the influence of glucose utilization on cholesterol biosynthesis were somewhat inconsistent. In 9 duplicate experiments, with glucose included in the incubation mixture, a stimulation of cholesterol biosynthesis of more than 20 per cent was found in 7 cases.

Not reported here are three earlier experiments with T5441 incubated in a Krebs Ringer bicarbonate buffer in which glucose was found without effect on cholesterogenesis. Addition of fructose, on the other hand, has always led to an increased cholesterogenesis.

In those cases where a stimulation of cholesterol synthesis was found it was never of the order of that seen in lipogenesis. Thus, it can be concluded that lipogenesis is more dependent upon carbohydrate utilization than cholesterogenesis in the tumours studied.

Besides the pathways leading to fatty acid and cholesterol, the two-carbon fragments derived from acetate-1-$C^{14}$ undergo oxidative degradation via the Krebs cycle yielding carbon dioxide. As a consequence of the latter reaction sequence, amino acid precursors (a.o. $\alpha$-ketoglutaric acid) are steadily being formed, leading to the synthesis of protein. When using radioacetate as the sole carbon source a similarity between the incorporation of tracer into carbon dioxide and protein can thus be expected. Such a parallel was found to exist roughly, as can be seen from the tables. It was reported in the previous paper (Emmelot and Bosch, 1955) that the hepatoma T15282 did not "take" on further transplantation. Experiments with a tumour of the last generation at hand revealed a lowering of all synthetic capacities as compared with earlier data. In this case glucose had a very significant stimulatory effect on both cholestero- and lipogenesis. As can be seen from the last experiment reported in Table IV, cholesterol synthesis and fatty acid synthesis were increased 300 and 1000 per cent respectively ; these were the highest stimulations found in the present work.

Another fact worth mentioning was the high specific activity of the respiratory carbon dioxide in both experiments with the hepatomas, as compared with other tumours. Thus, in this particular hepatoma, the oxidative pathway of acetate metabolism relative to endogenous $C_2$ oxidation is more pronounced than in the other three tumours, finding its equivalence in the labelling of the cholesterol and the protein of the hepatoma, but not to such extent in that of the fatty acids.

Neither the conversion of radioacetate to carbon dioxide nor its incorporation into protein, seems to be affected in a regular way by the simultaneous utilization of carbohydrate by the tumours. Although the stimulation of fatty acid synthesis by glucose addition to the tumour slices is of a lower magnitude than found by Hirsch, Baruch and Chaikoff (1954) studying the same effect in the rat mammary gland, the present data on the conversion of acetate-1-$C^{14}$ to carbon dioxide are at variance with theirs.

Hirsch, Baruch and Chaikoff (1954) found a significant lowering of the amount of acetate oxidized after addition of glucose, and concluded from this that the oxidative utilization of glucose shifted the two-carbon fragments, derived from acetate, from an oxidative path to one involving lipogenesis.

The present data do not allow one to draw this conclusion ; in fact, the stimulation in conversion of $C^{14}$ of acetate-1-$C^{14}$ to fatty acids, induced by carbohydrate, was never accompanied by a significant fall in the $C^{14}O_2$ recoveries. On the contrary an enhancement of acetate oxidation concomittant with the increase in lipogenesis was sometimes noted.

The differences between these findings may be due to the kind of tissues studied. Many normal tissues respond to the addition of carbohydrate by an increase in oxidation, whereas the tumour does so by glycolysis. Hence a shift of the two-carbon-$C^{14}$ intermediates to synthesis, due to an increase in glucose oxidation, need not be the actual course of an enhanced fatty acid synthesis and especially not in tumour tissue. Since only a small quantity of the C2-intermediates from unlabelled glucose reaches the Krebs cycle, because of the formation of lactic acid in the tumour tissue, it is evident that the stimulation which occurs must be due to glycolytic reactions. Although much work has already been done concerning the mechanism of the stimulatory action of glucose on fatty acid synthesis in normal tissues (Bloch, 1952), the exact cause of the effect is still obscure. The most plausible explanations are those which regard the formation of a metabolic " active form " of glycerol, the generation of adenosine triphosphate or both as determining the effect (Bloch, 1952). The first takes into account the formation of fat, since the absence of glycerol may be the rate limiting factor and the second envisages the energy requirements of the synthetic reactions concerned. Due to the active glycolysis encountered in the tumour tissues, both these substances can be generated in this reaction sequence and may be responsible for the stimulation of the lipid synthesis observed.

SUMMARY.

1. The incorporation of $C^{14}$ of acetate-1-$C^{14}$ in carbon dioxide, protein, cholesterol, and long-chain fatty acids by surviving tissue slices from a number of mouse tumour transplants has been studied in the presence and the absence of unlabelled fructose and glucose in the incubation mixture.

2. A marked stimulation of fatty acid synthesis was found after addition of glucose and somewhat less after addition of fructose.

3. Cholesterol synthesis was also found to be stimulated but to a smaller degree than fatty acid synthesis.

4. The stimulation in conversion of $C^{14}$ of acetate-1-$C^{14}$ to fatty acids and cholesterol, induced by the carbohydrates was never accompanied by a significant fall in the $C^{14}O_2$ recoveries.

5. It is concluded that the enhancement of fatty acid synthesis is directly related to the active glycolysis of the tumour tissues.

REFERENCES.

Artom, C.—(1953) *Ann. Rev. Biochem.*, **23**, 212.
Bloch, K.—(1952) *Ibid.*, **22**, 280.
Emmelot, P. and Bosch, L.—(1955) *Brit. J. Cancer*, **9**, 327.
Haugaard, E. S. and Stadie, W. C.—(1952) *J. biol. Chem.*, **199**, 741.
Hirsch, P. F., Baruch, H. and Chaikoff, I. L.—(1954) *Ibid.*, **210**, 785.
Lyon, I., Masri, M. S. and Chaikoff, I. L.—(1952) *Ibid.*, **196**, 25.
Masri, M. S., Lyon, I. and Chaikoff, I. L.—(1952), *Ibid.*, **197**, 621.
Wyshak, G. H. and Chaikoff, I. L.—(1953) *Ibid.*, **200**, 851.
Zamecnik, P. C., Frantz, I. D. Jr., Loftfield, R. B. and Stephenson, M. L.—(1948) *Ibid.*, **175**, 299.